# Supplementary material for: Striatal fast-spiking interneurons selectively modulate circuit output and are required for habitual behavior
Source: eLife. 2017 Sep 5;6:e26231. doi: 10.7554/eLife.26231 (PMC5584985; doi:10.7554/eLife.26231)
Supplement: Transparent reporting form [file elife-26231-transrepform.docx]

***eLife’s* transparent reporting form**

We encourage authors to provide detailed information *within their submission* to facilitate the interpretation and replication of experiments. If you have any questions, please contact us: [editorial@elifesciences.org](mailto:editorial@elifesciences.org).

**Sample-size estimation**

- You should state whether an appropriate sample size was computed when the study was being designed
- You should state the statistical method of sample size computation and any required assumptions
- If no explicit power analysis was used, you should describe how you decided what sample (replicate) size (number) to use

Please outline where this information can be found within the submission (e.g., page numbers or figure legends), or explain why this information doesn’t apply to your submission:

p. 24, lines 670 - 671

**Replicates**

- You should report how often each experiment was performed
- You should include a definition of biological versus technical replication
- The data obtained should be provided and sufficient information should be provided to indicate the number of independent biological and/or technical replicates
- If you encountered any outliers, you should describe how these were handled
- Criteria for exclusion/inclusion of data should be clearly stated
- High-throughput sequence data should be uploaded before submission, with a private link for reviewers provided (these are available from both GEO and ArrayExpress)

Please outline where this information can be found within the submission (e.g., page numbers or figure legends), or explain why this information doesn’t apply to your submission:

For information on how often experiments were repeated, see a detailed account of biological replicates (e.g. X cells from Y slices and Z mice) in Table 1.

Biological versus technical replicates are defined on p. 26, lines 778-779.

No data were excluded as outliers.

Statement that exclusion criteria were set prior to data unblinding can be found on p. 24, lines 670-671. Exact exclusion criteria are detailed in sections within Materials and methods, under Data analysis heading (start p. 24), corresponding to each type of experiment.

No high-throughput sequencing data included in study.

**Statistical reporting**

- Statistical analysis methods should be described and justified
- Raw data should be presented in figures whenever informative to do so (typically when N per group is less than 10)
- For each experiment, you should identify the statistical tests used, exact values of N, definitions of center, methods of multiple test correction, and dispersion and precision measures (e.g., mean, median, SD, SEM, confidence intervals; and, for the major substantive results, a measure of effect size (e.g., Pearson's r, Cohen's d)
- Report exact p-values wherever possible alongside the summary statistics and 95% confidence intervals. These should be reported for all key questions and not only when the p-value is less than 0.05.

Statistical tests used, and their justification based on normality, are described in the Statistics section of Materials and Methods on p. 26. Analysis methods not involving tests for significance, such as event detection in calcium imaging or electrophysiological experiments, are explained under the Data analysis heading in Materials and Methods (start p. 24), corresponding to each type of experiment.

Individual data points are shown in every figure where N is low enough to visually separate the points. Raw example traces of calcium imaging and electrophysiological recordings are also included throughout the manuscript.

Statistical details as listed in bullet #3 are included in figure legends. Typically, the test used is not explicitly mentioned in legends but measures of effect size are included and indicate the test used (e.g., F statistic indicates ANOVA). Details on exact tests used can be found in Statistics section of Materials and methods (p. 26).

Exact p values are now included in legends and text.

Please outline where this information can be found within the submission (e.g., page numbers or figure legends), or explain why this information doesn’t apply to your submission:

(For large datasets, or papers with a very large number of statistical tests, you may upload a single table file with tests, Ns, etc., with reference to page numbers in the manuscript.)

**Additional data files (“source data”)**

- We encourage you to upload relevant additional data files, such as numerical data that are represented as a graph in a figure, or as a summary table
- Where provided, these should be in the most useful format, and they can be uploaded as “Source data” files linked to a main figure or table
- Include model definition files including the full list of parameters used
- Include code used for data analysis (e.g., R, MatLab)
- Avoid stating that data files are “available upon request”

Please indicate the figures or tables for which source data files have been provided:

Figure 1D: Pre-post data are included for evoked calcium transients along with the parameters used for the Gaussian mixture model that separated SPNs into “low-firing” and “high-firing” clusters based on baseline responses. File: GMM_Parameters+SourceData.mat. Also included is the code used to generate the model: PrePostGMM.m. This code calls built-in functions that come with the MATLAB machine learning toolbox.
